# Tissue-specific and neural activity-regulated expression of human BDNF gene in BAC transgenic mice

**DOI:** 10.1186/1471-2202-10-68

**Published:** 2009-06-25

**Authors:** Indrek Koppel, Tamara Aid-Pavlidis, Kaur Jaanson, Mari Sepp, Priit Pruunsild, Kaia Palm, Tõnis Timmusk

**Affiliations:** 1 Department of Gene Technology, Tallinn University of Technology, Akadeemia tee 15, 12618 Tallinn, Estonia

## Abstract

**Background:**

Brain-derived neurotrophic factor (BDNF) is a small secreted protein that has important roles in the developing and adult nervous system. Altered expression or changes in the regulation of the BDNF gene have been implicated in a variety of human nervous system disorders. Although regulation of the rodent BDNF gene has been extensively investigated, *in vivo *studies regarding the human BDNF gene are largely limited to postmortem analysis. Bacterial artificial chromosome (BAC) transgenic mice harboring the human BDNF gene and its regulatory flanking sequences constitute a useful tool for studying human BDNF gene regulation and for identification of therapeutic compounds modulating BDNF expression.

**Results:**

In this study we have generated and analyzed BAC transgenic mice carrying 168 kb of the human BDNF locus modified such that BDNF coding sequence was replaced with the sequence of a fusion protein consisting of N-terminal BDNF and the enhanced green fluorescent protein (EGFP). The human BDNF-BAC construct containing all BDNF 5' exons preceded by different promoters recapitulated the expression of endogenous BDNF mRNA in the brain and several non-neural tissues of transgenic mice. All different 5' exon-specific BDNF-EGFP alternative transcripts were expressed from the transgenic human BDNF-BAC construct, resembling the expression of endogenous BDNF. Furthermore, BDNF-EGFP mRNA was induced upon treatment with kainic acid in a promotor-specific manner, similarly to that of the endogenous mouse BDNF mRNA.

**Conclusion:**

Genomic region covering 67 kb of human BDNF gene, 84 kb of upstream and 17 kb of downstream sequences is sufficient to drive tissue-specific and kainic acid-induced expression of the reporter gene in transgenic mice. The pattern of expression of the transgene is highly similar to BDNF gene expression in mouse and human. This is the first study to show that human BDNF gene is regulated by neural activity.

## Background

Brain-derived neurotrophic factor (BDNF) [1], a member of the neurotrophin family, promotes survival and differentiation of several neuronal populations during mammalian development [2,3]. In the adult central nervous system, BDNF acts as a regulator of activity-dependent neurotransmission and plasticity [4] and promotes survival of newborn hippocampal neurons [5]. BDNF has widespread expression in the developing and adult mammalian nervous system, its mRNA and protein levels rising dramatically in postnatal development [6-10]. In the adult, BDNF is also expressed in a number of non-neural tissues, with the highest levels of BDNF mRNA detected in thymus, heart and lung [11,12].

BDNF gene has a complex structure with multiple untranslated 5' exons alternatively spliced to one protein-coding 3' exon. The rat BDNF gene structure initially described to contain five exons [13] has been recently updated with a number of newly discovered exons for rodent [14,15] and human [16,17] BDNF. Untranslated 5' exons are linked with differentially regulated promoters directing tissue-specific expression of BDNF [13-17]. Furthermore, recently discovered BDNF antisense transcripts in human may exert additional control over BDNF transcription [16,17]. BDNF is a neural activity-dependent gene in rodents: various physiological stimuli induce its expression in neurons through excitatory neurotransmission-triggered calcium influx [18,19]. However, no data is available about activity-dependent transcription of the human BDNF gene in neurons, except one report showing that dopamine signaling increases the levels of BDNF exon IV transcripts in neuronally differentiated human embryonic teratocarcinoma NT2 cells [20].

Alterations in BDNF function have been associated with a variety of disorders of the nervous system [2]. As therapies modulating neurotrophic activity are being actively sought [21], it is of great importance to create model systems for studying the regulation of BDNF gene. BAC transgenic mice have proven useful in studying gene regulation as a) BAC clones are often long enough to contain all necessary DNA elements to recapitulate the expression patterns of endogenous genes independent of host genomic sequences flanking the transgene integration site and b) they can be easily modified with homologous recombination in *E. coli*, e.g. to introduce reporter genes under the control of promoters of interest [22]. BAC transgenes with EGFP reporter gene have been used for characterization of expression and regulatory regions of several neural genes [23-25]. Transgenic mice have been generated previously to study BDNF gene regulation *in vivo *[26,27]. Mouse lines carrying rat BDNF sequences of 10 kb range recapitulated BDNF expression only partially, suggesting that *cis*-acting regulatory elements necessary for accurate control of BDNF expression are located further away [26]. Recently, YAC-BDNF transgenic mice carrying 145 kb of human BDNF locus with BDNF coding sequence substituted for the EGFP reporter gene have been reported [27].

In this study we have generated BAC transgenic mice carrying human BDNF-EGFP fusion (hBDNF-EGFP) reporter gene under the control of 168 kb of human BDNF genomic sequences. C-terminal addition of EGFP to BDNF protein has been shown not to affect BDNF cellular localization, secretion and activation of its receptor TrkB in cultured neurons [28-30]. Therefore, to enable studying subcellular localization of the hBDNF-EGFP fusion protein *in vivo*, we specifically produced this fusion reporter gene construct. The aims of the study were to investigate a) expression of hBDNF-EGFP mRNA and protein in the brain and non-neural tissues and b) activity-dependent regulation of the hBDNF-EGFP transgene in the brain of the BAC transgenic mice.

## Results

### Generation of transgenic mice with 169 kb hBDNF-EGFP-BAC

**Figure 1:**
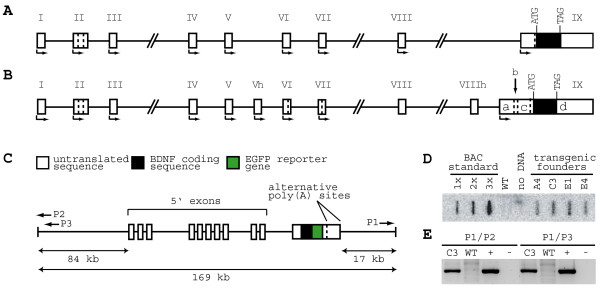
**Schematic drawings of rodent and human BDNF genes and the BAC transgenic construct used in this study**. Rodent (**A**) and human (**B**) BDNF gene structures. Rodent BDNF gene consists of a number of 5' exons (I-VIII) spliced together with a common protein-coding sequence in exon IX (transcriptional start sites are indicated with arrows). BDNF transcription can also start from exon IX introducing a unique 5' UTR sequence. Hatched lines indicate sites of alternative splicing. Although the human BDNF gene has a similar structure and splicing pattern, it has additional exons Vh and VIIIh, longer and more complexly spliced 5'UTR of exon IX. Furthermore, human BDNF exons VIII and VIIIh are not used as 5'exons, but are always spliced with exon V. For detailed description see [14,16]. (**C**) Schematic drawing of the modified BAC construct used in this study containing the human BDNF locus. EGFP reporter gene was inserted in-frame with the BDNF coding region before the BDNF stop codon creating a fused BDNF-EGFP open reading frame within 168 kb of human BDNF locus. Arrows P1-3 indicate PCR primers used for analysis of transgene integration. (**D**) Slot-blot hybridization analysis of transgene copy number in hBDNF-EGFP transgenic founder mice (A4, C3, E1 and E4). BAC standard contains hBDNF-EGFP-BAC DNA in amounts equivalent to 1–3 copies of transgene in the blotted genomic DNA. WT- wild type mouse DNA. (**E**) PCR analysis of genomic DNA from transgenic mouse line C3 with primers detecting tandem integration of hBDNF-EGFP-BAC constructs. WT – wild type mouse DNA as a negative control; (+) – circular hBDNF-EGFP-BAC DNA as a positive control; (-) – PCR without DNA as a negative control.

A 168 kb BAC clone extending 84 kb upstream and 17 kb downstream of human BDNF gene was used to generate human BDNF-EGFP reporter transgenic mice (see Materials and Methods and Figure 1A–C). Briefly, EGFP reporter gene was inserted in-frame with BDNF coding region replacing the BDNF stop codon (Figure 1C). Resulting hBDNF-EGFP fusion protein was expected to mimic subcellular localization of endogenous BDNF, allowing fine resolution of transgene expression. hBDNF-EGFP-BAC construct was tested for integrity using PCR and restriction analysis (data not shown). Transgenic mice were generated by pronuclear injection, yielding four transgenic founders (A4, E1, E4 and C3). All founders contained one to two transgene copies as estimated by slot-blot hybridization (Figure 1D). PCR analysis of C3 genomic DNA and sequencing of the PCR products revealed tandem integration of two transgene copies and confirmed the intactness of 5' and 3' end sequences of the integrated transgene (Figure 1E). Offspring was obtained from three founders and bred for several generations to generate transgenic mouse lines E1, E4 and C3.

### Expression of hBDNF-EGFP in transgenic mouse tissues

**Figure 2:**
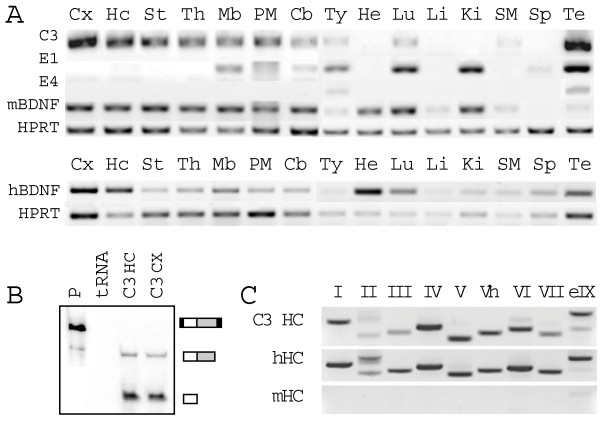
**hBDNF-EGFP mRNA expression in tissues of three transgenic mouse lines**. (**A**) RT-PCR analysis of hBDNF-EGFP mRNA expression in tissues of three transgenic BAC mouse lines – C3, E1, E4. mBDNF – mouse BDNF; hBDNF – human BDNF in human tissues; HPRT – reference gene hypoxanthine phosphoribosyltransferase. *Cx *– cortex; *Hc *– hippocampus; *St *– striatum; *Th *– thalamus; *Mb *– midbrain; *PM *– pons/medulla; *Cb *– cerebellum; *Ty *– thymus; *He *– heart; *Lu *– lung; *Li *– liver; *Ki *– kidney; *SM *– skeletal muscle; *Sp *– spleen; *Te *– testis. (**B**) Analysis of hBDNF-EGFP mRNA expression levels in C3 mouse brain by RNase protection assay. hBDNF-EGFP probe was used to determine both transgenic and endogenous BDNF mRNA levels as protein coding sequences of mouse and human BDNF share a high degree of similarity. *P *– probe without RNase; *tRNA *– yeast tRNA; *HC *– hippocampus; *CX *– cortex. On the right, black boxes denote vector-derived sequences, white boxes BDNF and gray boxes EGFP sequences. (**C**) Expression of alternative hBDNF-EGFP transcripts in C3 mouse hippocampus (HC), analyzed by RT-PCR. PCR primers used were specific for human BDNF transcripts as shown by control reactions with human (hHC) and mouse (mHC) hippocampal cDNA. eIX – transcript containing 5'-extended exon IX.

From three transgenic founder lines, C3 line showed pattern of expression of hBDNF-EGFP mRNAs that was highly similar to the expression of mouse endogenous BDNF (mBDNF) mRNA (Figure 2A). RT-PCR analysis revealed relatively high transgene expression in all brain regions of C3 mice, including cerebral cortex, hippocampus, striatum, thalamus, hypothalamus, midbrain, pons, medulla and cerebellum. In non-neural tissues, high levels of transgene mRNA were detected in testis, moderate levels in thymus and lung and low levels in skeletal muscle. BDNF mRNA is endogenously expressed in all these tissues both in mouse and human [14,16]; (Figure 2A). However, dissimilarly from mouse endogenous BDNF mRNA, hBDNF-EGFP mRNA was not detected in heart and kidney, where relatively high levels of mBDNF mRNA were detected. Low expression of hBDNF-EGFP transgene in the mouse kidney correlates with the finding that BDNF is expressed at low levels in human kidney [8,16].

In E1 mice, transgene expression recapitulated that of the endogenous BDNF mRNA in thymus, lung, kidney and testis, but not in other non-neural tissues that express BDNF. In the adult brain of E1 mice, transgene mRNA expression was detected in midbrain, cerebellum, pons and medulla at levels that were lower than in the respective brain regions of C3 mice. In E4 line, hBDNF-EGFP mRNA was detected only in testis and thymus (Figure 2A).

Expression of transgenic hBDNF-EGFP mRNA was further examined in different brain regions of C3 mice since this line largely recapitulated endogenous BDNF expression and expressed the transgene at the highest levels. Quantification of hBDNF-EGFP transcripts in C3 hippocampus and cortex using ribonuclease protection assay (RPA) revealed that transgene mRNA levels were about tenfold lower than endogenous mBDNF mRNA levels (Figure 2B). Analysis of transcription from the alternative human BDNF promoters in C3 mice confirmed the expression of all transcripts with different 5' exons described to date (exons I-IXe) both in hippocampus (Figure 2C) and cerebral cortex (data not shown).

**Figure 3:**
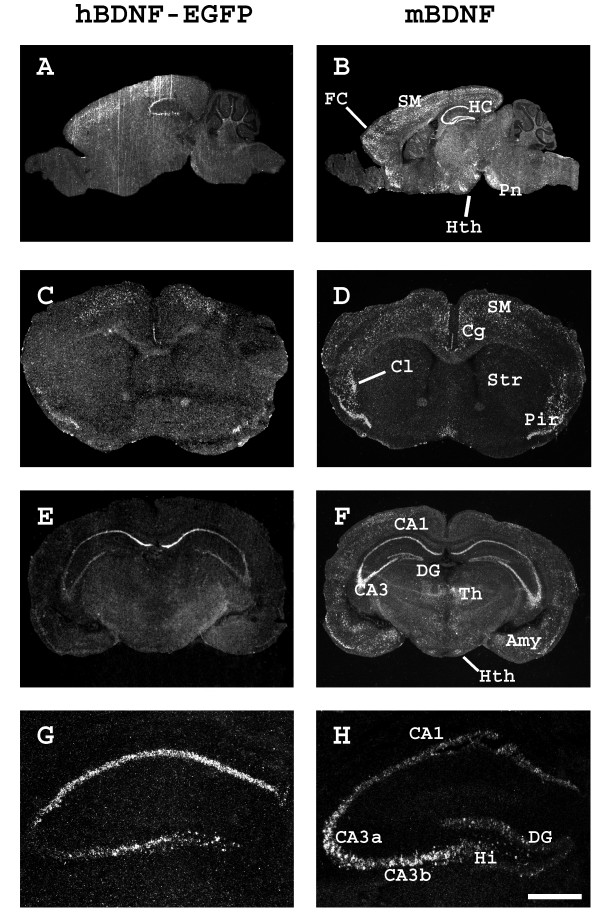
**Overlapping patterns of BAC-driven hBDNF-EGFP and mBDNF mRNA expression in C3 mouse brain**. *In situ *hybridization analysis, photoemulsion autoradiographs of 16 μm sagittal (**A**,**B**) and coronal (**C**-**H**) sections. (**C**) and (**D**) are sections taken at striatal level; (**E**) and (**F**) are sections taken at posterior hippocampal levels; (**G**) and (**H**) show enlarged hippocampal area (scale bar: 0,5 mm). *FC *– frontal cortex;*SM *– sensorimotor cortex; *HC *– hippocampus; *Pn *– pontine nuclei; *Hth *– hypothalamus; *Cg *– cingulate cortex; *Pir *– piriform cortex; *Cl *– claustrum; *Str *– striatum; *CA1, CA3 *– hippocampal subfields; *DG *– dentate gyrus of hippocampus; *Hi *– hilar area of dentate gyrus; *Th *– thalamus; *Amy *– amygdala.

**Figure 4:**
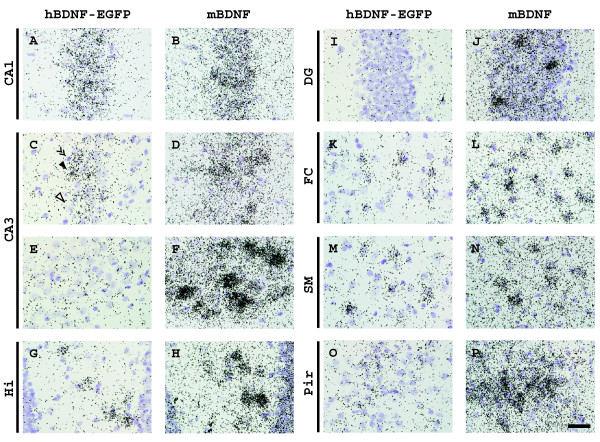
**Cellular expression of hBDNF-EGFP mRNA in adult C3 mouse brain**. *In situ *hybridization analysis, shown are bright-field autoradiographs of emulsion-dipped sections. Hybridization probes are indicated above the columns. Filled arrowhead indicates a neuron with strong labeling, empty arrowhead indicates a neuron with weak or absent labeling and double arrowheads indicate a glial cell showing no labeling. *CA1, CA3 *– hippocampal subfields; *DG *– dentate gyrus of hippocampus; *Hi *– hilar area of dentate gyrus; *FC *– frontal cortex; *SM *– sensorimotor cortex; *Pir *– piriform cortex. Scale bar: 20 μm.

*In situ *hybridization of C3 mice adult brain sections revealed hBDNF-EGFP mRNA expression in the hippocampus, particularly in the pyramidal neurons of CA1 and CA3 regions and in the polymorphic neurons in the hilus of the dentate gyrus, and also in several cortical areas, including neurons of frontal, sensorimotor and piriform cortex (Figure 3, 4). Endogenous mBDNF mRNA was detected in all brain areas where hBDNF-EGFP mRNA labeling was observed. However, hBDNF-EGFP labeling was absent or below the detection limit of our *in situ *hybridization assay in several areas expressing mBDNF mRNA, e.g. claustrum, amygdala, thalamic, hypothalamic and pontine nuclei. Furthermore, *in situ *hybridization showed differential expression of hBDNF and mBDNF in cortical and hippocampal subfields. While mBDNF mRNA was expressed at high levels throughout the cerebral cortex, hBDNF-EGFP labeling was more prominent in the frontal cortex and in the sensorimotor area extending along the longitudinal fissure (Figure 3C, D and Figure 4K–N). In the hippocampus, hBDNF-EGFP labeling was observed over the CA1 and hilar subfields and part of the CA3 subfield (CA3b in Figure 3G, H and Figure 4C, D), mimicking the pattern of expression of endogenous mBDNF mRNA. On the other hand, hBDNF-EGFP mRNA was expressed at considerably lower levels in the part of CA3 subfield that showed high levels of mBDNF mRNA expression (CA3a in Figure 3G, H and Figure 4E, F). In addition, no hBDNF-EGFP labeling was detected in the granule neurons of dentate gyrus where endogenous mBDNF mRNA was highly expressed (Figure 3G, H and Figure 4I, J).

**Figure 5:**
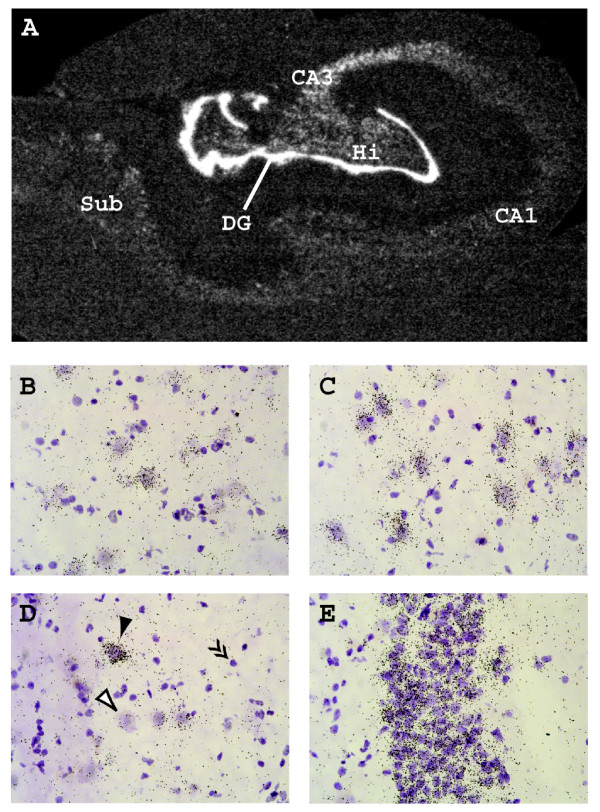
**Expression of BDNF mRNA in the human hippocampus**. (**A**) *In situ *hybridization autoradiograph of a 16 μm coronal section.*DG *– granular layer of dentate gyrus; *Hi *– hilar area of dentate gyrus; *Sub *– subiculum; *CA1, CA3 *– hippocampal subfields. (**B-E**) High magnification bright-field photomicrographs of hematoxylin-counterstained neurons in subfields CA1 (**B**) and CA3 (**C**), the hilus (**D**) and granular layer of dentate gyrus (**E**). Filled arrowhead indicates a neuron with strong labeling, empty arrowhead indicates a neuron with weak or absent labeling and double arrowheads indicate a glial cell showing no labeling.

Since the BDNF gene in the transgenic construct was of human origin, we also analyzed the expression of BDNF in the human hippocampus using *in situ *hybridization. In agreement with earlier findings [31,32], our results showed that the highest levels of hBDNF mRNA were present in the granule cells of dentate gyrus, whereas other hippocampal regions showed relatively weaker expression (Figure 5). However, strong hBDNF labeling was detected over majority of CA3 and CA1 neurons using high magnification (Figure 5B, C), indicating that these areas show much weaker signal in the dark-field image partly because of the scarcity of neuronal cell bodies in the CA1 and CA3 subfields of the human hippocampus.

Next we examined expression of hBDNF-EGFP fusion protein across tissues in C3 mice. No EGFP fluorescence was observed in brain sections or cultured primary embryonic (E18) hippocampal neurons. In addition, hBDNF-EGFP protein was not detected in the hippocampus, cortex and testis by Western blot analysis with anti-EGFP or anti-BDNF antibodies (data not shown). hBDNF-EGFP open reading frame in C3 genomic DNA was analyzed for possible mutations by sequencing and was found to be intact. Together with mRNA expression data these results suggest that hBDNF-EGFP protein was either not translated in the brain and testis of C3 mice or was expressed at levels below the detection limits of our methods.

### Kainic acid induces hBDNF-EGFP mRNA expression in transgenic mouse brain

**Figure 6:**
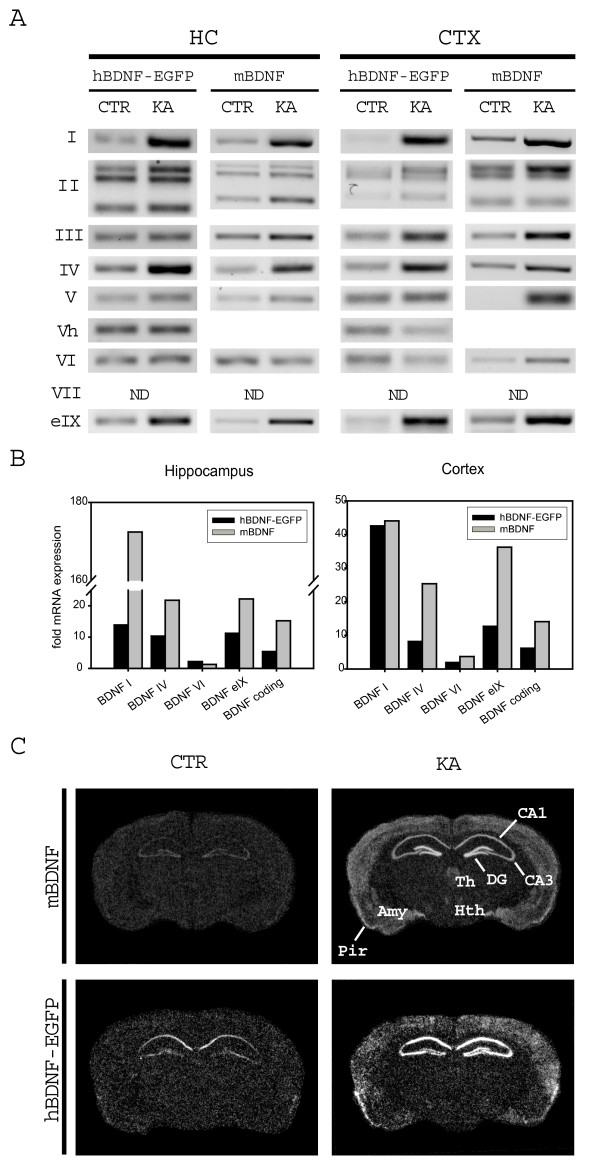
**Kainic acid (30 mg/kg) induces transgenic hBDNF-EGFP mRNA expression in brains of C3 line transgenic mice**. (**A**) Induction of alternatively spliced hBDNF-EGFP transcripts in C3 mouse hippocampus (HC) and cerebral cortex (CTX), analyzed with RT-PCR. mBDNF – mouse transcripts; ND – not determined; KA – kainic acid treated mice; CTR – control mice. Three BDNF-II bands correspond to alternatively spliced transcripts. (**B**) Quantitative real-time RT-PCR analysis of selected BDNF transcripts, normalized to HPRT1 levels and expressed as fold difference relative to mRNA levels in untreated mice. (**C**) *In situ *hybridization autoradiographs of C3 mouse coronal brain sections. *Pir *– piriform cortex; *CA1, CA3 *– hippocampal subfields; *DG *– dentate gyrus of hippocampus; *Hi *– hilar area of dentate gyrus; *Th *– thalamus; *Hth *– hypothalamus; *Amy *– amygdala.

Kainic acid (KA), agonist of the KA subtype ionotropic glutamate receptor, has been shown to induce BDNF mRNA levels in adult rodent hippocampus and cerebral cortex [13,19,33,34]. KA induction of transgenic hBDNF-EGFP transcripts in the hippocampus and cerebral cortex of C3 mice largely followed the induction pattern of endogenous mBDNF transcripts (Figure 6A). KA markedly upregulated both endogenous mouse and transgenic hBDNF-EGFP transcripts containing exons I, IV and 5'-extended exon IX (eIX) in the hippocampus and cortex. hBDNF-EGFP and mBDNF mRNAs containing other 5' exons were induced to a lesser extent. Of note, recently described human-specific exon Vh-containing transcripts were not induced by KA in transgenic mice in the context of 169 kb hBDNF-EGFP BAC construct (Figure 6A).

Levels of BDNF transcripts showing the most robust induction by kainic acid were analyzed further using quantitative real-time RT-PCR analysis (Figure 6B). Transgenic hBDNF-EGFP exon I, exon IV and 5'-extended exon IX transcripts, and total hBDNF-EGFP mRNA were potently induced in both hippocampus and cortex following 3 hours of kainate treatment, similarly to respective endogenous mBDNF mRNAs. Exon VI-containing hBDNF-EGFP and endogenous mBDNF transcripts showed no induction, which is consistent with previous findings [13,14,33].

*In situ *hybidization analysis showed marked induction of transgenic hBDNF-EGFP mRNA by KA in the pyramidal neurons of CA1-CA3 layers, in the hilar region of hippocampus and also in the layers II – VI of cerebral cortex (Figure 6C). Importantly, kainic acid induced transgene expression also in the granular layer of dentate gyrus of hippocampus, whereas control animals did not show any detectable expression in this area. Endogenous mBDNF was induced in the same neuronal populations, suggesting that the 169 kb hBDNF-EGFP BAC construct contains all the regulatory elements that mediate kainic acid induction. We also examined expression of the hBDNF-EGFP protein in the brains of kainic acid treated C3 mice by direct EGFP fluorescence and Western blot analysis but no fusion protein was detected (data not shown).

## Discussion

In this study, BAC transgenic mice carrying 168 kb of the human BDNF locus and encoding human BDNF-EGFP fusion protein were generated and analyzed. Out of three analyzed founder lines, one line (C3) largely recapitulated human BDNF mRNA expression in the brain, thymus, lung, skeletal muscle and testis. Founder line E1 mimicked human BDNF mRNA expression in some brain regions, and also in thymus, lung and kidney. Founder line E4 expressed transgene only in the thymus and testis. These results showed that although all three founder lines expressed hBDNF-EGFP mRNA at different levels, the 169 kb BAC construct, carrying 67 kb of human BDNF gene, 84 kb of 5' and 17 kb of 3' sequences, contains regulatory elements necessary for hBDNF mRNA expression in many brain regions and non-neural tissues. However, integration site-dependent expression of transgene in different founder lines suggests that the BAC construct may not contain necessary insulator elements to protect it from the influence of genomic regions flanking the transgene integration site. It has been shown for many genes that insulators can functionally isolate neighboring genes and block their interactions [35].

In several non-neural tissues, the 169 kb hBDNF-EGFP BAC recapitulated endogenous expression of both mouse and human BDNF. Transgenic mRNA was expressed in the thymus and testis in three mouse lines, expression in the lung was seen in two lines and only one line expressed hBDNF-EGFP in the kidney and skeletal muscle. All these tissues have been shown to express BDNF both in mouse and human [7,14,16]. Of note, all three founder lines expressed relatively high levels of hBDNF-EGFP in adult testis, in contrast to the very low expression levels of endogenous mBDNF in the testis. This transgene expression pattern can be explained by human origin of the BDNF gene as relatively high levels of BDNF mRNA, comparable to the levels in the brain, have been detected in the human testis [16]. In the adult human testis, expression of BDNF and its receptor TrkB has been reported in Leydig, Sertoli and germ cells [36], while in the adult mouse testis, BDNF expression has been detected in Sertoli cells and expression of its receptor TrkB in germ cells [37]. These findings indicate differences in BDNF expression between human and mouse and are further supported by the present study. On the other hand, none of the founder lines expressed hBDNF mRNA in the heart, a tissue with high levels of BDNF expression both in human and rodents [8,11,12,14]. This suggests that distinct heart-specific regulatory elements are located outside of the genomic DNA fragment that was included in the BAC construct.

Detailed analysis of hBDNF-EGFP expression in the C3 mouse brain by *in situ *hybridization showed that the transgene mimicked mBDNF expression in many neuron populations, including neurons of the CA1-CA3 and hilar regions of the hippocampus and the cerebral cortex. However, hBDNF-EGFP failed to recapitulate endogenous BDNF expression in several neuron populations, including the granule cells of dentate gyrus of hippocampus where BDNF mRNA is expressed both in human and rodents. hBDNF-EGFP expression was detected in all analyzed brain regions by RT-PCR, but not by *in situ *hybridization, indicating that transgene mRNA levels in several brain structures were below the detection limit of our *in situ *hybridization analysis.

BDNF transcription is regulated by neuronal activity through calcium-mediated pathways [18,38]. Systemic treatment of rodents with kainic acid (KA) has been used to model activity-dependent induction of BDNF mRNA in the nervous system [13,19,33,34]. Here we show that KA differentially induced alternative hBDNF-EGFP transcripts in the cortex and hippocampus (for comparison with mouse and rat see Table 1). Pronounced induction of transgenic hBDNF-EGFP transcripts containing exons I, IV, and 5'-extended exon IX (eIX), moderate induction of transcripts containing exons II, III and absence of induction of transcripts containing exon VI is consistent with the induction pattern of respective BDNF mRNAs in mouse and rat [13,14,33]. To our knowledge, this is the first time to report neural activity-dependent regulation of the human BDNF gene *in vivo*. Real-time PCR showed that total transgenic mRNA, as well as transcripts containing exons I, IV and 5'-extended exon IX were induced to a lesser extent than the respective endogenous mBDNF mRNAs. This is consistent with earlier results reported for shorter rat BDNF transgenes [26] and could be caused by increased stability of transgenic BDNF-reporter mRNAs as compared to the mouse endogenous BDNF mRNAs. Alternatively, the absence of important regulatory elements in the transgenic construct may underlie the reduced induction of the transgene by kainic acid. *In situ *hybridization analysis of KA-treated C3 mouse brains showed induction of hBDNF-EGFP mRNAs in several neuronal populations where endogenous BDNF mRNA levels were also increased. These results show that, similarly to rodent BDNF, expression of the human BDNF gene is induced by neural activity and that regulatory elements mediating the induction are included in the 168 kb of the human BDNF locus contained in the BAC transgene. Several regulatory elements located in the rat BDNF proximal promoter IV and the transcription factors mediating activity-dependent activation of this promoter have previously been characterized [39]. Among these elements, CRE (cAMP-response element) was found to be the most important for Ca^2+^-mediated activation of rodent BDNF promoter IV [40-42]. However, the respective regulatory elements and transcription factors responsible for the activity-dependent regulation of the human BDNF gene have not been characterized. Transgenic mice described here can be used to study the regulation of human BDNF gene *in vivo *using a variety of methods successfully applied in the studies of rodent BDNF [39].

**Table 1:** Regulation of human, mouse and rat BDNF exon-specific mRNAs by kainic acid in the hippocampus and cerebral cortex.

	human^1^		mouse^2^		rat^3^
exon	HC	CTX	HC	CTX	HC

I	**	**	**	**	**

II	*	*	*	*	*

III	*-	*	*	*	-

IV	**	**	**	**	**

V	*	*	*	**	**

Vh	-	-	X	X	X

VI	-	-	-	-	-

VII	ND	ND	ND	ND	**

VIII	X	X	ND	ND	*

eIX	**	**	**	**	**

- no induction; * weak induction; ** strong induction; ND – not determined; X – transcript containing this exon as the 5' exon does not exist in this organism; ^1,2 ^based on data from the present study; ^3 ^based on data from [14]; HC – hippocampus; CTX – cerebral cortex.

Previously, transgenic mice carrying shorter fragments of the BDNF locus have been generated and characterized [26,27]. Mice expressing the CAT reporter gene under the control of 9 kb of rat BDNF genomic sequences covering promoters I-III or promoters IV-VI showed relatively high CAT activity in most tissues and brain regions expressing endogenous BDNF mRNA. *In situ *hybridization analysis showed that these constructs carrying either BDNF promoters I-III or IV-VI were able to drive CAT mRNA expression in adult rat brain in a pattern largely overlapping with mouse BDNF mRNA expression. Nevertheless, recapitulation of endogenous BDNF expression had a number of shortcomings in these transgenes: both constructs were not expressed or were expressed at low levels in the dentate granule cells and granule cells of cerebellum; BDNF IV-VI did not mimic BDNF expression in the heart; both constructs displayed relatively high reporter activity in the striatum where rat BDNF is virtually not expressed [43]. It was assumed that these transgenic constructs lacked important regulatory elements, which could be present in a much longer gene fragment than the BAC clone used here. Although BAC transgenic mouse lines generated in this study showed improved recapitulation of expression as compared to that of the BDNF-CAT transgenic mice [26], we could not detect transgene expression in several tissues and neuron populations that express endogenous BDNF mRNA.

A recent study reported generation of human BDNF-EGFP transgenic mice using a 145 kb YAC clone including 45 kb of 5' and 33 kb of 3' flanking sequences of hBDNF gene with the protein coding sequence partially replaced with EGFP reporter gene [27]. Three out of five transgenic founder lines obtained in that study expressed transgenic mRNA in the brain and only one of these showed expression of transgenic hBDNF transcripts containing exons IV and VI in the heart. Out of three lines analyzed, EGFP fluorescence was detected in the brain of only one line, specifically in the claustrum, intermediate layer of parietal cortex, pyramidal cell layer of CA3 hippocampal subfield and a population of neurons in the granule cell layer of the dentate gyrus. However, EGFP fluorescence was not detected in other cortical neuron populations and in the CA1 region of hippocampus where rodent and also human BDNF mRNA are expressed [27]. Differences in the tissue- and neuron-specific expression of transgenic hBDNF-EGFP mRNA and protein between the study by Guillemot et al. [27] and this study can be explained with different lengths of the BDNF gene-flanking genomic regions in the transgenic constructs used: the hBDNF-BAC used in the present study contained 39 kb longer 5' and 16 kb shorter 3' genomic regions of hBDNF gene than the reported hBDNF-YAC construct [27]. In addition, part of BDNF coding sequence had been replaced with EGFP reporter gene in the hBDNF-YAC transgene [27], possibly removing *cis*-elements with regulatory function. In contrast to the present study, hBDNF-YAC transgenic mRNA expression was not analyzed in different brain regions and expression of transgenic mRNAs containing exons III, V, Vh, VII and 5'-extended exon IX was not analyzed. More detailed comparison of hBDNF-EGFP expression in the two hBDNF transgenic mouse models would allow narrowing down genomic regions containing enhancer elements for tissue-specific expression of human BDNF. For example, on the basis of current data it can be hypothesized that a *cis*-element promoting heart-specific expression of hBDNF mRNA is located within the 3' terminal 16 kb of hBDNF-YAC construct (17–33 kb downstream of the hBDNF gene; chr11:27,600,000–27,616,000; UCSC Genome Browser, Mar 2006 Assembly). Recently, a BDNF regulatory locus has been discovered 850 kb upstream of the human and mouse BDNF genes that causes obesity, cognitive impairment and hyperactivity when disrupted [44,45]. Therefore, it is possible that in addition to regulatory elements included in the hBDNF-BAC of this study and the hBDNF-YAC described before [27], others can be found hundreds of kilobases away from the BDNF gene.

EGFP reporter gene has been successfully used to visualize BAC-driven expression of neural genes in a number of studies [23-25]. In the BAC construct that was used to generate transgenic mice in the present study, EGFP reporter gene was fused C-terminally with the human BDNF coding sequence to allow detailed characterization of human BDNF expression in the nervous system. Unfortunately, we could not detect EGFP protein in the brain of C3 mice neither with fluorescence microscopy nor with Western blot analysis. This could be explained with low levels of hBDNF-EGFP protein expressed in the C3 mouse brain as transgenic hBDNF-EGFP mRNA levels were about tenfold lower than these of endogenous BDNF. It is also possible that founder mice with higher levels of BDNF-EGFP expression died during embryonic development due to overactivation of BDNF receptor TrkB. This hypothesis is supported by a study showing that embryonic overexpression of BDNF from nestin promoter results in gross abnormalities in brain architecture and perinatal death [46]. Although the hBDNF-EGFP fusion protein can be expressed in cultured cells *in vitro *[28-30], it is conceivable that it is not translated or has poor translatability and/or stability when expressed in transgenic mice *in vivo*.

## Conclusion

Human genomic region covering 67 kb of the BDNF gene, 84 kb of upstream and 17 kb of downstream sequences is able to drive tissue-specific and kainic acid-induced expression of reporter gene in transgenic mice that largely overlaps with BDNF gene expression and regulation in mouse and human. This is the first study to directly show that human BDNF gene is regulated by neural activity. The BDNF-BAC transgenic mice are useful for studying the transcription regulation of human BDNF gene *in vivo*. In addition, these mice could be used for screening therapeutic agents modulating human BDNF transcription.

## Methods

### Generation of transgenic mice

BAC clone (RP11-651M4) containing the human BDNF locus [GenBank:AC087446.13] was purchased from Chori BACPAC Resources (USA). Red^®^/ET^® ^homologous recombination in *E. coli *(Counter-Selection BAC Modification Kit, Gene Bridges GmbH, Germany) was used to delete BDNF stop codon and to insert EGFP reporter gene with the linker sequence (CGG GAT CCA CCG GTC GCC ACC) into the 3' end of BDNF. For sequences of primers used for insert synthesis see Table 2. Modified BAC was tested for the absence of rearrangements using EcoRV restriction analysis and pulsed field gel electrophoresis. Integrity of the hBDNF-EGFP reading frame was confirmed by sequencing. In order to validate the reporter activity, BAC DNA was purified using the Large Construct Purification Kit (Qiagen, USA) and transfected into COS-7 cells using DEAE-dextran mediated transfection system [47]. Five days after transfection EGFP expression and distribution in COS-7 cells was visualized using fluorescence microscopy (Eclipse 80i upright microscope, Nikon).

**Table 2:** PCR primers used in this study

**Primer/application**	**Sequence**
**BAC modification**	

hBDNFcod_rpsL_neo_s	5' GGATAGACACTTCTTGTGTATGTACATTGACCATTAAA AGGGGAAGATAGGGCCTGGTGATGATGGCGGGATCG 3'

hBDNF_rpsL_neo_as	5'AATAGATAATTTTTGTCTCAATATAATCTAATCTATACAACATAAATCCATCAGAAGAACTCGTCAAGAAGG 3'

hBDNFcod_linker_EGFP_s	5' TAAGGATAGACACTTCTTGTGTATGTACATTGACCAT TAAAAGGGGAAGACGGGATCCACCGGTCGCCACCATGGTGAGCAAGGGCGAGGAGCTG 3'

hBDNF_EGFP_as	5' AATAGATAATTTTTGTCTCAATATAATCTAATCTATAC AACATAAATCCATTACTTGTACAGCTCGTCCATGCCGA 3'

**genotyping/slot-blot hybridization/expression analysis**	

hBDNF_s	GTACGTGCGGGCCCTTACCATGGATAGC

EGFP_as	TGGTGCAGATGAACTTCAGGGTCAGC

**expression analysis**	

mBDNF_s	GTATGTTCGGGCCCTTACTATGGATAGC

mBDNF_as	AAGTTGTGCGCAAATGACTGTTTC

HPRT1_s	CTTTGCTGACCTGCTGGATTAC

HPRT1_as	GTCCTTTTCACCAGCAAGCTTG

hBDNF_I_s	GATGCCAGTTGCTTTGTCTTCTGTAG

hBDNF_II_s	GGGCGATAGGAGTCCATTCAGCACC

hBDNF_III_s	AGTTTCGGGCGCTGGCTTAGAG

hBDNF_IV_s	GCTGCAGAACAGAAGGAGTACA

hBDNF_V_s	TCGCGTTCGCAAGCTCCGTAGTG

hBDNF_Vh_s	GGCTGGAACACCCCTCGAA

hBDNF_VI_s	GGCTTTAATGAGACACCCACCGC

hBDNF_VII_s	GAACTGAAAGGGTCTGCGACACTCT

hBDNF_IXb_s	GCTGCTAAAGTGGGAAGAAGG

hBDNF_IX_as1	GTCCTCATCCAACAGCTCTTCTATC

hBDNF_IX_as2 (with VII_s)	GAAGTGTACAAGTCCGCGTCCTTA

**expression analyis (qPCR)**	

EGFPq_s	CAGAAGAACGGCATCAAGGTG

EGFPq_as	TGGGTGCTCAGGTAGTGGTTG

hBDNFq_I_s	CAGCATCTGTTGGGGAGACGAGA

hBDNFq_IV_s	GAAGTCTTTCCCGGAGCAGCT

hBDNFq_VI_s	ATCGGAACCACGATGTGACT

hBDNFq_IXc_s	AACCTTGACCCTGCAGAATGGCCT

hBDNFq_IX_as1 (with I, IV_s)	ATGGGGGCAGCCTTCATGCA

hBDNFq_IX_as2 (with VI_s)	ACCTTGTCCTCGGATGTTTG

hBDNFq_IX_as3 (with IXc_s)	GATGGTCATCACTCTTCTCACCT

mBDNFq_I_s	TTGAAGCTTTGCGGATATTGCG

mBDNFq_IV_s	GAAATATATAGTAAGAGTCTAGAACCTTG

mBDNFq_VI_s	GCTTTGTGTGGACCCTGAGTTC

mBDNFq_IXa_s	GGACTATGCTGCTGACTTGAAAGGA

mBDNFq_IX_as1 (with I, IV, VIs)	AAGTTGCCTTGTCCGTGGAC

mBDNFq_IX_as2 (with IXa_s)	GAGTAAACGGTTTCTAAGCAAGTG

mBDNFq_coding_s	GGCCCAACGAAGAAAACCAT

mBDNFq_coding_s	AGCATCACCCGGGAAGTGT

HPRT1q_s	CAGTCCCAGCGTCGTGATTA

HPRT1q_as	AGCAAGTCTTTCAGTCCTGTC

**transgene integrity**	

pBACe3.6_SP6 (5'end)	TATTTAGGTGACACTATAG

rp11_5'_as (5'end)	GGACAACAGACCCAAGGAGA

rp11_3'_s (3'end)	GTAGGGTGTCTGGGTTGGTG

pBACe3.6_T7 (3'end)	TAATACGACTCACTATAGGG

**transgene tandem integration**	

rp11_3'_s (P1)	GTAGGGTGTCTGGGTTGGTG

pBACe_11326_s (P2)	CGGTTACGGTTGAGTAATAAATGGATG

pBACe_11365_s (P3)	GGGGCACATTTCATTACCTCTTTCTC

hEGFP-BDNF BAC DNA was purified for microinjection by alkaline lysis and linearized with PI-SceI enzyme (NEB, USA). Restriction solution was separated in low-melt agarose gel (Fermentas, Lithuania) using CHEF-DR II Pulsed Field Electrophoresis System (Bio-Rad, USA). Linearized BAC DNA was excised from the gel and purified from agarose using Gelase enzyme (NEB, USA). Transgenic mice were generated by pronuclear injection of linearized hBDNF-EGFP-BAC into CBA × C57Bl/6 mouse pronuclei in the Karolinska Center for Transgene Technologies (Sweden). Founder mice carrying the BAC transgene were identified by PCR analysis of genomic DNA. Transgene copy number was analyzed by slot-blot hybridization of genomic DNA with a [α-^32^P]dCTP-labeled probe generated with HexaLabel DNA Labeling Kit (Fermentas, Lithuania) using pEGFP-N1 (Clontech, USA) plasmid as a template. Genomic DNA of the C3 mouse founder line was analyzed by PCR for the presence of 5' and 3' ends of the linearized transgene. Tandem insertion of transgene into the C3 line genomic DNA was analyzed by PCR with primers pBACe_11326_s or pBACe_11365_s in combination with rp11_3'_s (see Table 2) and sequencing of the PCR product. All animal experiments were performed in agreement with the local Ethical Committee of Animal Experimentation.

### Cell culture, antibodies and animal experiments

African green monkey kidney fibroblast COS-7 cells were grown in DMEM with 10% fetal calf serum and antibiotics. Primary neuronal cultures from embryonic day 18 cerebral cortex were prepared as described [48]. For Western blots and immunohistochemistry the following antibodies were used: mouse anti-GFP monoclonal antibodies (Roche Applied Science), mouse anti-GFP monoclonal antibodies (Clontech, USA); rabbit anti-BDNF (Santa Cruz Biotechnology, USA). For kainic acid treatment, adult mice weighing 20–25 g were injected intraperitoneally with 30 mg/kg of kainic acid or 1× PBS. 3 hours later mice were decapitated, hippocampus and cortex dissected, frozen on dry ice and stored at -70°C. For *in situ *hybridization whole brains were embedded in Shandon Cryomatrix™ (Thermo Fisher Scientific, USA). Four kainic acid-treated C3 mice and two control mice were used for quantitative RT-PCR analysis of total hBDNF-EGFP mRNA expression in the cerebral cortex and hippocampus. Total hBDNF-EGFP mRNA was induced 2,5–6 fold in the hippocampus of kainic acid-treated C3 mice and the mouse displaying highest induction of hBDNF-EGFP and mBDNF mRNA was analyzed further with RT-PCR for expression of exon-specific transcripts. Five kainic acid-treated C3 mice and two control mice were used for *in situ *hybridization analysis and the mouse showing highest induction of hBDNF-EGFP and mBDNF mRNA was further analyzed in more detail.

### RT-PCR

Total RNA was isolated from mouse and human tissues using TRI reagent (Ambion, USA). All experiments with human tissues were approved by the local Ethical Committee for Medical Research. Two mice from each transgenic line were analyzed for tissue-specific expression of hBDNF-EGFP mRNA in brain regions and non-neural tissues and they showed identical transgene expression pattern. RNA was treated with DNase (DNA-*free*, Ambion, USA) following manufacturer's instructions and five micrograms of total RNA was used for cDNA synthesis with oligo-dT primer (Microsynth, Switzerland) and SuperScript III reverse transcriptase (Invitrogen, USA). PCR amplification was carried out with HotFire DNA polymerase (Solis Biodyne, Estonia) according to the manufacturer's instructions. Quantitative real-time PCR was performed on a LightCycler 2.0 instrument (Roche Applied Science) using qPCR Core kit for SYBR^® ^Green I No ROX (Eurogentec, Belgium). Melting curve analysis was carried out at the end of cycling to confirm amplification of a single PCR product. All qPCR reactions were performed in triplicate and normalized to hypoxanthin phosphoribosyltransferase 1 (HPRT1) mRNA levels.

### Ribonuclease protection assay

For cRNA synthesis 624 bp BDNF-EGFP fragment containing 452 bp of BDNF, 21 bp linker sequence and 151 bp of EGFP sequence was amplified with PCR from modified BAC clone RP11-651M4 and cloned into pBluescript SK+ vector (Stratagene, USA). [α-^32^P]UTP-labeled cRNA probe was *in vitro *transcribed from linearized plasmid template using MAXIscript Kit and T3 polymerase (Ambion, USA). 10 μg of total RNA and 2.5 ×10^5 ^CPM of radiolabeled probe were used for RPA hybridization and the assay was performed with the RPA III Kit from Ambion as suggested by the manufacturer. The protected fragments were separated in 4% acrylamid-urea gel and detected autoradiographically using BioRad Molecular Imager FX.

### In situ hybridization

cRNA probe complementary to the coding region was used to mouse BDNF mRNA and probe complementary to EGFP was used to detect hBDNF mRNA. Probes were synthesized from DNA fragments subcloned into pCR4-TOPO vector (Invitrogen, USA). [α-^35^S]UTP-labeled probes were generated with MAXIScript In Vitro Transcription Kit (Ambion, USA) using linearized DNA template and T3 or T7 RNA polymerase. 16 μm sections of fresh-frozen C3 mouse brain were processed according to the protocol described in [13]. Slides were exposed to either BioMax MR X-ray film for one week or NTB-2 photoemulsion for 2 months, developed with D19 developer and fixed with a general-purpose fixer (all from Eastman Kodak, USA). Slides exposed to NTB-2 were counterstained with hematoxylin (Vector Laboratories Inc., USA).

## Authors' contributions

IK bred and analyzed the transgenic mice, performed in situ hybridization and RT-PCR analysis. TAP prepared the BAC-BDNF-EGFP construct, carried out transfection experiments and initial characterization of the transgenic mice. KJ performed transgene integration analysis, RT-PCR experiments and contributed to the breeding of founder lines. MS performed RNase protection assay, Western blot analysis and fluorescence microscopy. PP contributed to the initial characterization of the transgenic mice, cultured embryonic neurons and performed in situ hybridization analysis of BDNF mRNA expression in human hippocampus. KP conceived and coordinated the preparation of the transgenic construct. TT conceived and coordinated the study. IK and TT co-wrote the manuscript, all authors contributed to the analysis of the results and preparation of the manuscript. All authors read and approved the final manuscript.
